# Municipal Dwellings for Working Men

**Published:** 1896-03-14

**Authors:** 


### The Hospital

An Institutional Journal of

The Medical Sciences and Hospital Administration, with

AN EXTRA NURSING SUPPLEMENT.

Vol. XIX.—No. 494.] [March 14, 1896.

### Municipal Dwellings for Working Men.

Seeing the enormous sums paid every year in rent by working men, it is not surprising that many people should look upon the acquisition of the freehold of his dwelling as the first step to success on the part of an artizan. Our knowledge, however, of the actual condition of affairs leads us to a very different conclusion: to the belief, in fact, that to the working man a reasonable rent, combined with liberty, is a far less onerous burden than ownership, combined as it always is with restricted opportunities of disposing of his labour, together with responsibility for rates, repairs, and sanitation. This is the very centre of the problem. Whatever else may be true in regard to the relations between employer and employed, it is certain that the rate of wages is influenced, among other things, by the mobility of labour, and that wherever workers are tied to any particular locality by the ownership of their homes there the employer will be able to obtain their services at a rate determined, not alone by the value of their labour, but also by their attachment to their freeholds.

So much for economics. But there are other causes which make it undesirable for working people to become owners of their houses, especially in towns. On the death of the bread-winner the house, bought by the scrapings and savings of years, is apt to be found unsuitable for the remainder of the family, and thus it has to be sold, often at great loss. What the owner may have saved in rent his heirs too frequently lose in realising the property, and so the purchase of his dwelling by a workman is far from always being the good investment that some imagine.

From a public health point of view also the separate ownership of individual town houses is bad. It is nearly impossible to induce the weekly wage - earner to put by sufficient for the repair of his house, and the demands made on him by the sanitary authorities in regard to drainage and other matters are apt to be resented in every way. In fact, it is a great hardship that a man who has lived a life of carefulness for the sake of escaping rent should have to pay almost as much in repairs as he has thus saved. There is no class of houses so fertile in sanitary troubles as that owned by impecunious people. The demands of sanitarians seem to them arbitrary, and to frugal people eating off broken platters it may well seem insulting irony to demand large sums to replace a cracked closet pan. Yet these things have to be enforced, and so great have been the practical sanitary difficulties connected with individual ownership of cottage property, that some of the large companies for supplying workmen's dwellings, which started with the intention of encouraging workpeople to become owners of their own homes, have for this reason had to buy back such houses as had been disposed of.

What this item of repairs means may be gathered from the report of the Peabody trustees, who give the cost of repairs during 1895 as nearly half the total net gains of the year, and we may be quite sure that such work done by retail jobbing builders for individual owners would cost much more even than this.

From a careful consideration, then, of all sides of the question, we can have no doubt that working men are likely to be best lodged where their houses are owned in large blocks by people—be they corporations or individuals—who can employ their own workmen for the doing of repairs, and are accessible to the pressure of the sanitary authorities, and where the rent is kept at a reasonable level by competition with a standard set by the municipal authorities.

In regard to the erection of workmen's dwellings by municipalities, the example of the Peabody Donation Fund should be borne in mind. The sum given by Mr. Peabody was: In 1862, £150,000; in 1866, £100,000; in 1868, £100,000; and received by bequest from him in 1873, £150,000; making a total of £500,000; to which has been added money received for rent and interest, £669,338 16s. 8d.; making the total fund on December 31st last £1,169,338 16s. 8d. That sums of money given between 1862 and 1873, and invested in workmen's dwellings, should have more than doubled themselves by 1895, is enough to show that such investments are remunerative, and to cut the ground from beneath the feet of those who protest against municipalities spending money on workmen's dwellings. It is clear enough that, even under municipal management, they ought to return more than the interest on the money spent; and if they are so managed as to do that, while the ratepayers will have no cause for complaint, the private owner of cottage property need have no anxiety, for in the difference between his own economies and the more costly municipal management will lie his profits.

We do not suggest that municipalities should embark on the vast enterprise of housing their entire working-class population, but they might well supply a competitive standard of accommodation so far as can be done without loss. The Peabody Fund, of course, was a gift, and the entire yearly profits go back into the undertaking for the erection of still more houses. Any fund, however, advanced by a municipality would have first to pay interest on itself, but all beyond that should, as in the Peabody Fund, go to the further development of the scheme. The plan would, in fact, be automatic. Wherever rents were unduly high profits would be made, which would all go to create more buildings; while where rents were low, and demand for houses small, the profits would also be small, and the scheme would not extend. By itself setting a sanitary standard, and by rigidly enforcing it upon all owners of house property, a municipality will be doing much towards what surely is within its province, viz., ensuring to each dweller within its gates the possibility of living a healthy life; and if it will follow the example of the Peabody Trustees it should do this at no loss. What should be done with the very lowest stratum of the population—the residuum, as it has been called—is another matter, to which we may return later.

